# Compressive loading at the end plate directly regulates flow and deformation of the basivertebral vein: an analytical study

**DOI:** 10.1186/1749-799X-1-18

**Published:** 2006-12-27

**Authors:** Ming-Long Yeh, Michael H Heggeness, Hsiang-Ho Chen, Jennifer Jassawalla, Zong-Ping Luo

**Affiliations:** 1 Department of Orthopedic Surgery, Baylor College of Medicine, Houston, TX, USA; 2 Biomedical Engineering Institute, National Cheng-Kung University, Tainan, Taiwan, ROC

## Abstract

**Background:**

Metastatic diseases and infections frequently involve the spine. This is the result of seeding of the vertebral body by tumor cells or bacteria delivered by venous blood from Batson's plexus, which is hypothesized to enter the vertebral body via the epidural veins. Isolated spinal segments deform significantly at the bony end plate when under compression. This deformation could cause a volume change of the vertebral body and may be accompanied by retrograde flow of venous blood. To date, this process has not been investigated quantitatively. The purpose of this study was to determine the volume changes of the vertebral body and basivertebral vein for a vertebral body under compression.

**Methods:**

A three-dimensional finite element mesh model of the L4 segment with both adjacent discs was modified from a 3-D computed tomography scan image. An octagon representing the basivertebral vein was introduced into the center of the vertebral body in the model. Four compressive orientations (1500 N) were applied on the top disc. The volume change of the vertebral body model and the basivertebral vein were then computed.

**Results:**

The volume change of the vertebral body was about 0.1 cm^3 ^(16.3% of the basivertebral vein) for the four loading conditions. The maximum cross-sectional area reductions of the basivertebral vein and volume reduction were 1.54% and 1.02%, for uniform compression.

**Conclusion:**

Our study quantified the small but significant volume change of a modeled vertebral body and cross-sectional areas and that of the basivertebral vein, due to the inward bulging of the end plate under compression. This volume change could initiate the reverse flow of blood from the epidural venous system and cause seeding of tumors or bacterial cells.

## Background

It is well-known that the venous drainage network for the bony vertebral column is unique [1-4]. The caliber of the basivertebral veins of the vertebral body is far out of proportion to similar venous drainage systems for other bones of the body, including large weight-bearing bones [1,5]. Venous blood drains from the basivertebral vein into the epidural system via the central vascular foramen of the vertebral body. This blood then enters the highly compliant epidural system which is continuous with the valveless network of Batson's plexus [1].

Metastatic disease and infections frequently involve the spine. This is widely believed to be the result of seeding of the vertebral body by tumor cells or bacteria delivered to the vertebral body by venous blood from Batson's plexus, which is hypothesized to enter the vertebral body via the epidural veins [1-3,6-9]. It has been proposed that during daily activities such as straining, coughing, or lifting with the upper extremity, blood is not only prevented from entering the thoracicoabdominal cavity, it is actually squeezed out of the cavity. Tumors and abscesses of the thoracicoabdominal viscera and retroperitoneal space connected with the Batson's plexus may therefore be shed out from the cavity and distributed anywhere along the vertebral system of veins [1,7]. If this widely held belief is correct, then some retrograde flow of blood within the vertebral body must take place to allow intraosseus entry of bacteria or tumors. The large caliber of the basivertebral vein and the flexible epidural system therefore presents an appropriate anatomic accommodation for the volumes of venous blood which must enter and exit the vertebral body with each cycle of loading, allowing for possible intermittent retrograde flow. We speculate that large end plate deformation under normal physiological loading observed from experimental studies [10,11] could cause a volume change of the vertebral body. This volume change is accommodated by antegrade and retrograde flow of venous blood in and out of the valveless epidural vein system, which is continuous with Batson's plexus.

Previous works have shown that isolated spinal motion segments, when subjected to axial load, deform significantly at the bony end plate [10-17]. If endplate deflection of the vertebral body is a significant mechanism for axial compression in the spine, the volume change within the vertebral bones may occur concomitantly. The spine is constantly subjected to intermittent compressive loading during daily activities. Since compressive motion of the spine occurs naturally in a rapid manner, this action requires some means for the rapid accommodation of these volume changes. This may be accomplished by the flow of venous blood. To date, the volume change of the venous vessel has not been investigated.

We hypothesize that volume within the vertebral body is directly regulated by the compressive loading applied on the end plate. The volume changes within the vertebra under compressive loading could be accommodated by the blood volume and flow of the basivertebral vein with antegrade and retrograde flow of venous blood in and out of the valveless epidural vein system. The volume change of the vertebral body directly altering the total volume of the intraosseous blood vessel could be one of the driving forces for this reverse flow of the vertebral venous system.

In this study, we examined the volume change of the vertebral body under different loading conditions using finite element analysis, and the cross-sectional area and volume changes of the basivertebral vein were calculated to verify the hypothesis.

## Methods

**Figure 1:**
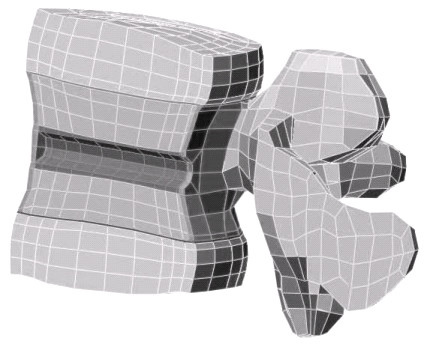
Finite element model of L4 vertebra under uniform compression at the top of the disc. The spinal geometry is symmetrical to the medial plane, so only half of the structure is needed to model uniform, anterior and posterior compression conditions.

A three-dimensional finite element mesh model (ALGOR Inc, Pittsburgh, PA) of the adult L4 segment with both adjacent discs modified from a 3-D computed tomography (CT) scan image of an adult spine was downloaded from Finite Element Meshes Repository of the International Society of Biomechanics (Fig. 1). The spinal geometry is symmetrical to the medial plane, so only half of the structure is necessary for the models under uniform, anterior and posterior compression conditions. An octagon representing the basivertebral vein was introduced into the center of the vertebral body. The geometry of the vertebral body was divided into cortical shell, hard and soft cancellous bone, and cartilaginous end plate. The isotropic material properties (elastic modulus and Poisson's ratio) of the cortical shell (14.5 GPa, ν = 0.3), hard (227 MPa, ν = 0.3) and soft (113 MPa, ν = 0.3) cancellous bone, cartilaginous end plate (24.5 MPa, ν = 0.3), and disc annulus (6.5 MPa, ν = 0.3) were applied to the geometry [13]. Because of the highly porous structure of cancellous bone, the Poisson's ratio could be smaller than 0.3. Therefore, Poisson's ratio for cancellous bone (0.1) was also used.

The end plate deflection of the vertebral segment was very sensitive to the accuracy of the thickness of the cortical shell because the modulus difference between cortical and cancellous bone was 50 to 100 times. The thickness of the cortical shell from the original CT scan image might not well represent its real value. For confirming the model in this study, the end plate deflection of the vertebral segment under uniform compression was matched to the previous experimental data by deliberately adjusting the thickness of the cortical shell.

Four loading conditions were simulated with a compressive force of 1500 N applied on the top disc: uniform, lateral portion, anterior portion, and posterior portion. The bottom disc was fixed. The deformations of the end plate, vertebral body, and basivertebral vein were calculated. The change of the vertebral body volume and cross-sectional areas along the basivertebral vein and its volume were computed.

## Results

The deflection, or inward bulging, at the center of the vertebral endplate was 0.185 mm when the thickness of the bony endplates was modified from 1 mm to 0.4 mm. It was compared to the studies using experimental measurements under the same loading condition with good agreement [10,12].

The volume decreases in the vertebral body were 0.102 cm^3 ^(0.35%), 0.110 cm^3 ^(0.37%), 0.092 cm^3 ^(0.31%) and 0.107 cm^3 ^(0.36%) for uniform, anterior, posterior, and lateral loading, respectively (Table 1). The volume change increased to 0.130 cm^3^, 0.142 cm^3^, 0.114 cm^3^, and 0.138 cm^3 ^for uniform, anterior, posterior, and lateral loading, respectively, as Poisson's ratio for cancellous bone (0.1) was used (Table 1).

**Table 1:** Vertebral body volume changes under different loading and Poisson's ratio (0.3, 0.1) for cancellous bones.

	(ν = 0.3)		(ν = 0.1)	
Loading	cm^3^	%	cm^3^	%

Uniform	-0.102	-0.35%	-0.130	-0.44%
Anterior	-0.110	-0.37%	-0.142	-0.48%
Posterior	-0.092	-0.31%	-0.114	-0.39%
Lateral	-0.107	-0.36%	-0.138	-0.47%

**Figure 2:**
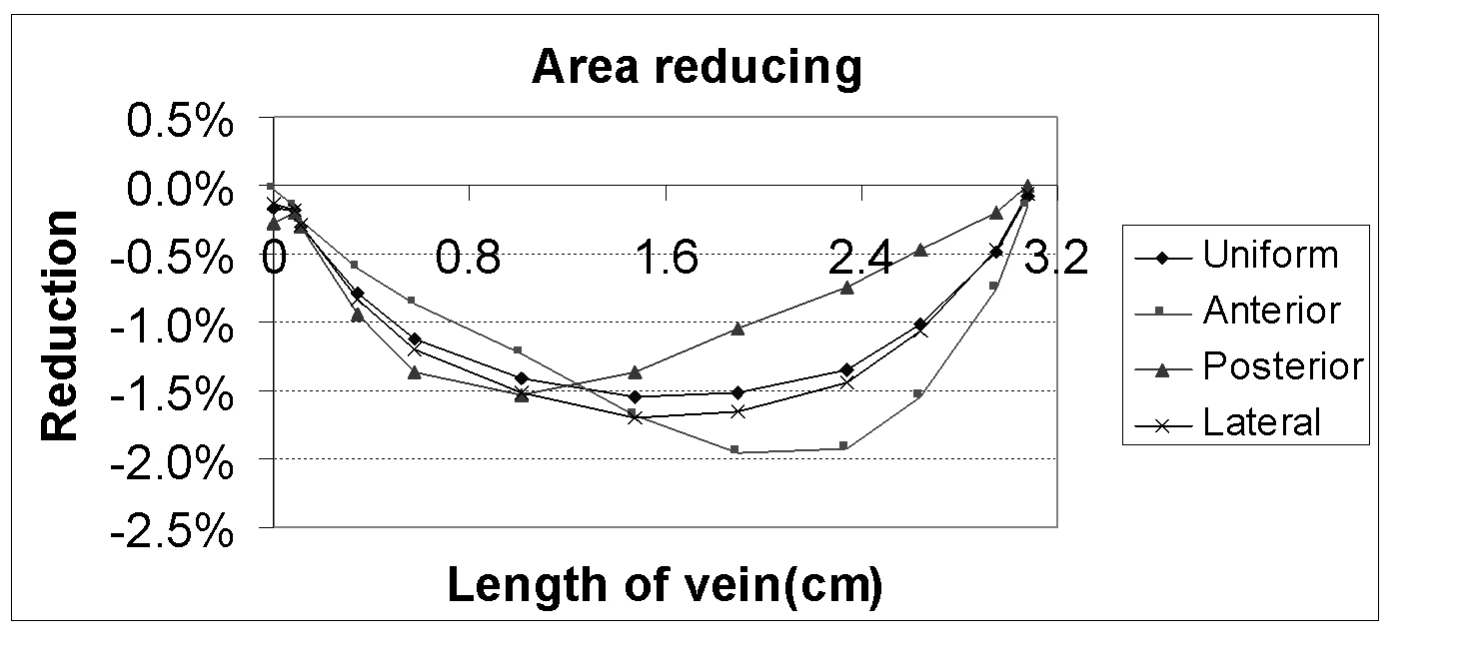
Cross-sectional area reduction along the basivertebral vein.

The cross-sectional area changes of the basivertebral vein were affected by the loading conditions and locations of the vein. The cross-sectional area deformed differently at various segments of the basivertebral vein (Fig. 2). The maximum cross-sectional area reductions of the vein near the center of the vertebral body were 1.54%, 1.96%, 1.53% and 1.69% of the original cross-sectional areas for uniform, anterior, posterior and lateral loading, respectively (Table 2). The reductions of the cross-sectional areas at the posterior exit of the basivertebral vein were less than 0.28% in all loading conditions.

**Table 2:** The cross-sectional area and volume changes at the basivertebral vein under different loading conditions.

Loading	Area		Volume	
	
	Center	Posterior	cm^3^	%
Uniform	-1.54%	-0.16%	-0.0064	-1.02%
Anterior	-1.96%	-0.03%	-0.0071	-1.14%
Posterior	-1.53%	-0.28%	-0.0051	-0.82%
Lateral	-1.69%	-0.14%	-0.0068	-1.08%

The volume changes in the basivertebral vein were influenced by the loading conditions. The original volume of the basivertebral vein in this model was 0.62655 cm^3^. The volume decrements of the basivertebral vein were 1.02%, 1.14%, 0.82%, and 1.08% of the original volume of the basivertebral vein for uniform, anterior, posterior and lateral loading, respectively (Table 2).

## Discussion

Our study found that the volume decrease for the vertebral body was larger than 0.1 cm^3^, and the reduction ratio of the volume of the basivertebral vein was about 1% when the vertebral body was under 1500 N axial compression. Although the percentage volume reduction of the basivertebral vein was not large, the percentage of the vertebral body volume change to the volume of the basivertebral vein in this model could be up to 17.5% (0.11 cm^3 ^to 0.62655 cm^3^) for anterior compression. The deformation of the vertebral body could be accommodated by the blood flow in and out of its basivertebral venous system. Conversely, the vertebral body and basivertebral vein would expand when the loading was removed. The cross-sectional area change of the basivertebral vein at the posterior exit, less than 0.28%, was significantly less than at the center (1.53% to 1.96%) for various loading conditions.

The deformation for posterior loading was less significant because the compression was resisted by the spinous process. This implied that it is easier to create a blood vessel volume change during spine flexion than during extension.

Previous studies have shown significant changes in vertebral body pressure according to posture and position, with increased intervertebral pressures reproduced by postures that increased axial load, a phenomenon that is not seen in weight-bearing long bones [5,18,19]. Axial compression at the vertebral body causing significant end plate deflection was observed by experimental measurement [10,12]. This study further computed the vertebral body and venous blood vessel volume change under the axial loading that caused similar end plate deformation. The direct accommodation of the volume change of the basivertebral vein for the vertebral body under compression would be a flow of venous blood. The volume of the basivertebral vein only slightly changed (1%); however, the volume change of the vertebral body compared to the basivertebral vein was significant (17.5%). The volume change of the vertebral body under compression could result from the compressibility of the solid phase of cortical and cancellous bones themselves; however, the volume change could also be accompanied by intraosseous flow. The pressure applied at the end plate is over 100 times higher than intraosseous pressure. The nature of the highly porous structure of cancellous bone causes most of the accompanying deformation of the vertebral body by squeezing the space among the solid bony materials, i.e. constricting the flow of blood inside it. The volume change of the vertebral body under uniform compression was about 16.3% of the volume of the basivertebral vein; thus, almost 1/6 of the blood inside the basivertebral vein was turned over under single compressive loading. Combining the deformation of the vertebral body and the basivertebral vein, the spinous venous system would function as a sucking device to retract the blood into the spine when the compression on the vertebral body was quickly released. This result would explain the mystery of a high incidence of tumor or bacterial seeding of the spine on the basis of a pumping action on the venous blood, powered by reversible endplate deflection.

The Poisson's ratio used in the model directly affected the compressibility of the material. The volume change of the vertebral body under uniform compression for Poisson's ratio (0.3) of cancellous bone used in this model was 0.102 cm^3^. It increased to 0.130 cm^3 ^when the Poisson's ratio was 0.1. The *in vivo *actual Poisson's ratio of the bony material is difficult to calculate because of the inclusion of the liquid phase of blood and fluid and their irrigation within the vertebral body. However, 0.3 Poisson's ratio should be a conservative estimation. The volume change of the vertebral body under uniform compression was about 16.3% of the volume of the basivertebral vein using Poisson's ratio (0.3) for cancellous bone. This percentage of volume change for vertebra under rapid loading and unloading should be the driving force to generate the retrograde flow of basivertebral blood flow.

One of the advantages of this finite element method is the ability to calculate the profile of the cross-sectional area change along the basivertebral vein. The direct assessment of the reduction of the basivertebral vein cross-sectional area by experimentation remains challenging. In a previous finite element study, the vertebral body of the two-dimensional model was assumed to be axially symmetrical, and the venous vessel was not included [13]. In our study, the profile of the cross-sectional area change along the entire basivertebral vein was calculated, as was the volume change of the vertebral blood vessel. The deformation distribution along the blood vessel matched the bulging at the end plate. The blood vessels near the surface of the vertebral body are surrounded by a cortical bony shell, so the cross-sectional area change of the basivertebral vein at its surface exit was much less than at its center (Table 2). Although it would be easier to measure the change of the cross-sectional area of the basivertebral vein at the bony surface under loading, using this value to predict the volume change of the venous vessel would underestimate the actual volume changes within the vertebral body.

To our knowledge, this is the first theoretical simulation to elucidate the correlation between the loading at the spinal body and the high incidence of seeding of tumor cells. Limitations exist in some aspects of the model: (1) the geometry and material properties of the vertebral body and (2) the shape of the spinal venous system [1]. The human spine is a complex biological structure which consists of alternating cortical and cancellous bony components. The elastic modulus of cortical bone is about 100 times higher than cancellous bone. The deformation of the bony segment is very sensitive to the accuracy of the dimension of the cortical end plate. The geometry in this model was obtained from a 3-D CT image; however, the thickness of the cortical bone in the original CT image might not precisely represent the true dimension of the bony end plate in individual human patients. The effects of osteoporosis on this system, for example, are not known. However, osteoporosis would be predicted to increase the deflections studied herein. The finite element model in this study was carefully evaluated before its use.

End plate deflection has been measured by cadaveric studies yielding results consistent with those of this study [10,12]. The thickness of the cortical shell in our study was carefully adjusted from 1 mm to 0.4 mm to match the end plate deformation from previous studies.

The blood vessel was modeled using an octagonal shape inserted through the vertebral body. Although the basivertebral vein is only present at a portion of the vertebral body, the combination of the upstream small veins and capillaries might deform equivalently to a single large blood vessel inside the vertebral body. In the future, more detailed geometry representing the circulatory system inside the vertebral body and the amount of pressure change in response to the blood vessel volume change needs to be studied.

In summary, our study quantified the small but significant volume change of the vertebral body and the cross-sectional areas and volume change of the basivertebral vein due to the inward bulging of the end plate under compression. This high ratio of volume change of the vertebral body to the volume of the blood vessel could initiate reverse blood flow from the epidural venous system and cause seeding of vertebral bone tumors or bacterial cells.

## Competing interests

The author(s) declare that they have no competing interests.

## Authors' contributions

MLY assembled the computer models and images, collected the data and compiled the manuscript. MHH contributed clinical expertise and input regarding the pathology of spinal tumors and physiology of the spinal venous drainage. HHC provided biomechanical expertise regarding the experimental design. JJ assisted with the computer modeling and data collection. ZPL assisted with the research design, data analysis and writing of the manuscript. All authors read and approved the final manuscript.
